# Balancing caution and ınnovation: exploring the potential of large language models in critical decision-making

**DOI:** 10.1186/s13054-023-04447-0

**Published:** 2023-05-04

**Authors:** Izzet Turkalp Akbasli, Benan Bayrakci

**Affiliations:** 1 Department of Pediatrics, Faculty of Medicine, Hacettepe University, Ankara, Turkey; 2 The Center for Life Support Practice and Research, Hacettepe University, Ankara, Turkey; 3 Department of Pediatric Intesive Care Medicine, Hacettepe University, Ankara, Turkey

To the Editor,

We recently read the thought-provoking paper by Azamfirei et al. [1], which delved into the limitations and ethical implications of using ChatGPT in critical decision-making processes. While we appreciate the authors' concerns and the importance of exercising caution when using language models like ChatGPT, we would like to highlight some alternative perspectives on this rapidly evolving technology.

To begin, we agree with the authors that new technologies should not be blindly adopted without proper understanding and evaluation. It is indeed crucial to be aware of the strengths and limitations of tools like ChatGPT, as well as their potential impact on various fields. However, it is also important to consider the remarkable advancements that have been made in recent years and the beneficial applications of language models.

For instance, following two major earthquakes in Turkey, OpenAI's GPT model [2] was employed to identify survivors' locations, as reported on Twitter. As a result, nearly thousand of people were rescued from the wreckage. This example demonstrates that when used appropriately and with human supervision, language models like ChatGPT can play a significant role in disaster management, public health, and other critical areas.

Furthermore, the authors' comparison of ChatGPT to a self-driving system navigating a rocket to low earth orbit offers a valuable perspective. While it is true that we should not use a tool designed for one purpose in an entirely different context, it is also essential to recognize the potential of these models to be adapted and improved for specific tasks. The development of specialized systems for summarizing scientific articles, for example, is not far-fetched and can significantly benefit researchers and practitioners across disciplines [3].

It is worth noting that the adoption of technology often follows a pattern of initial resistance, gradual acceptance, and eventual obsolescence as newer, better solutions emerge [4]. A prime example of this can be seen in the 1988 protest actions by mathematics teachers in the USA, who sought to ban the use of calculators in primary schools, as well as Fig. 1. [5]. Fast forward to the present day, the use of calculators has become far less prevalent, particularly in educational settings, as more advanced devices have rendered them almost obsolete.

**Fig. 1:**
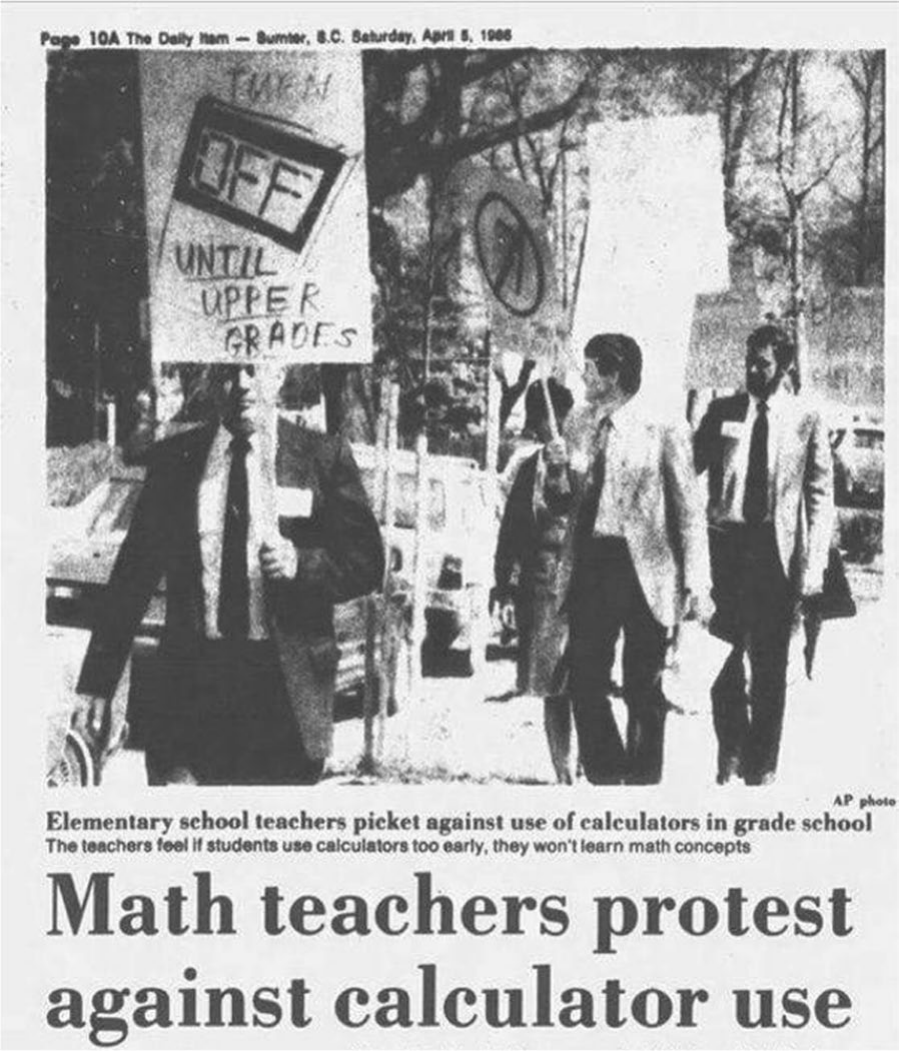
Teachers protesting against the early use of calculators in elementary schools, stating that it may hinder students' understanding of mathematical concepts [5]

In conclusion, we would like to express my gratitude to Azamfirei et al. for raising essential questions and concerns about the use of language models like ChatGPT. As technology advances at an accelerating pace, it is our collective responsibility to ensure that we understand, evaluate, and harness these tools for the betterment of society. By engaging in constructive discussions like the one initiated by the authors, we can work together to strike a balance between caution and innovation, ultimately making the most of the potential offered by emerging technologies.

## Data Availability

Not applicable, as no datasets were used in the creation of this manuscript.
